# An Improved Method for Estimating Core Size Distributions of Magnetic Nanoparticles via Magnetization Harmonics

**DOI:** 10.3390/nano10091623

**Published:** 2020-08-19

**Authors:** Yi Sun, Na Ye, Dandan Wang, Zhongzhou Du, Shi Bai, Takashi Yoshida

**Affiliations:** 1 School of Computer and Communication Engineering, Zhengzhou University of Light Industry, Zhengzhou 450001, China; sunyi3064@gmail.com (Y.S.); wangdandan0221@gmail.com (D.W.); 2 Department of Electrical and Electronic Engineering, Kyushu University, Fukuoka 819-0395, Japan; t_yoshi@ees.kyushu-u.ac.jp; 3 School of Information Science and Engineering, Shenyang University of Technology, Shenyang 110870, China; stworldyy@gmail.com

**Keywords:** magnetic nanoparticle, magnetization harmonics, *M*–*H* curve, core size distribution

## Abstract

The core size distribution is an important physical characteristic of magnetic nanoparticles (MNPs) because it seriously affects biomedical and biological applications. In this study, we proposed an improved method for estimating the distributions, which optimizes the excitation frequency based on AC susceptibility to avoid the effects of Brownian relaxation. Moreover, the first, third, and fifth magnetization harmonics under different excitation field strengths are used for estimating core size distributions to avoid measuring higher harmonics. The experiment results show that the improved AC harmonic method can accurately and quickly estimate the distribution of large core sizes compared with the method of static magnetization (*M*–*H*) curves, which is a competitive advantage in MNP immunoassays.

## 1. Introduction

In recent years, magnetic nanoparticles (MNPs) have become increasingly important in biosensor technology and biomedical applications, such as magnetic immunoassays, magnetic particle imaging (MPI), and hyperthermia cancer treatments [1,2,3,4,5,6,7,8]. The core size distributions of MNPs play an important role in governing performance in these applications. In immunoassays that use magnetic markers and sensors, the marker consists of coated MNPs coupled to an antibody. The binding reaction between an antigen and its antibody is detected by measuring the magnetic signal from the marker [1,2,3]. The core size distribution of MNPs can be wide in practical markers, and it is thus important to evaluate the marker parameters and magnetic properties. Furthermore, the applications in MPI and magnetic hyperthermia have been extensively studied, and they use MNP magnetizations under alternating magnetic fields [4,5,6,7]. The magnetizations depend on magnetic parameters such as core diameters and core size distributions. The time resolution and measurement accuracy are the main indexes in the estimation of core size distributions [8,9,10].

A fast and accurate method for estimating core size distributions is needed to optimize MNP applications. The common method for estimating the distributions is transmission electron microscopy (TEM). However, TEM delivers only local information on the sizes and shapes of MNPs rather than that of whole sample, and it is difficult to distinguish between particles once they agglomerate. Static magnetization (*M*–*H*) curves are very useful tools for estimating the core size distributions [11,12,13,14]. Berkov [14] discretized the equation for magnetization and obtained a numerical matrix related to a function for particle size distribution. The distribution may then be obtained by solving the ill-posed matrix equations. However, the magnetization signal at a low DC excitation field is very small because it is usually detected via an inductive pickup coil. As a result, the accuracy of the estimated distributions of MNPs with large core sizes will degrade. They dominate the magnetization at low excitation fields, and the magnetization signal is routinely used in biomedical applications. One solution to overcome this problem is the use of a high-density MNP sample. In this case, however, magnetic dipole interactions should be considered and can lead to complex magnetization models that are very time consuming. Biederer [15] used the spectrum of MNPs under an AC excitation field to estimate particle size distributions. However, the effect of rotational relaxation at excitation frequencies up to 25 kHz could lead to errors between experiment data and the model, resulting in errors in the estimation of the core size distribution. Ludwig [16] employed all the available magnetization harmonics of MNPs under a single excitation field strength to quantify binary and ternary mixtures of different particles, rather than the full core size distribution of MNPs. Because the amplitudes of the harmonics decrease with increasing harmonic number, their use under a single excitation field strength could limit the resolving power of the core size diameters. Moreover, the magnetization response of small particles is linear; thus, they may not have higher harmonics. Meanwhile, the higher harmonic signals are weak and result in measurement errors.

Regarding the issue above, we innovatively proposed an improved method for estimating the core size distribution of MNPs, in which only the primary magnetization harmonics (first, third, and fifth) under different excitation field strengths were used to estimate the core size distributions, to avoid measuring higher harmonics. Moreover, the excitation frequency was based on experimental AC susceptibilities to avoid the effect of Brownian relaxation. The simulation and experimental results show that the MNP core size distributions can be quickly and accurately obtained by solving the matrix equation for harmonic amplitudes. This will be significant for biosensor technology and biomedical applications.

## 2. Models and Methods

The magnetization of MNPs exposed to a low-frequency AC excitation field, *H_ac_* = *H*sin(*ωt*), can be described by the Langevin function [12,17,18,19]. Here, *H* is the amplitude of AC excitation field, *w* = 2*πf* is the angular frequency, and *f* is the frequency of the AC excitation field. The ensemble magnetization *M* can be described as follows:

(1) $$M\left(H_{ac}\right)=\frac{M_{s}}{V_{T}}\int_{D_{\min}}^{D_{\max}}n\left(D\right)V\left(D\right)L\left(\xi \right)dD,$$

with

(2) L(ξ)=coth(ξ)−1ξ,

(3) $$\xi =\frac{\mu_{0}mH_{ac}}{k_{B}T},\;\mathrm{and}\;m=\frac{\pi }{6}D^{3}M_{s},$$

where *L*(*x*) is the Langevin function, *ξ* is the ratio of external field energy to thermal energy, *n*(*D*) is the number of magnetic nanoparticles with core size diameter *D*, *V*(*D*) is the volume of magnetic nanoparticles with core size diameter *D*, *V_T_* is the total volume of the MNPs, *D*_min_ and *D*_max_ are the minimum and maximum, *k_B_* is Boltzmann’s constant, *T* is the absolute temperature, *M_s_* is the saturation magnetization, *m* is the magnetic moment, and *µ*_0_ is the permeability of the vacuum.

To obtain a numerical solution of the integral in Equation (1), *D* is given by discrete values and *M* can be rewritten as:

(4) $$M\left(H_{ac}\right)=\frac{M_{s}}{V_{T}}\sum_{k=1}^{K}n(D_{k})V(D_{k})L(\mu_{0}m_{k}H_{ac}/k_{B}T)\Delta D,$$

where *K* denotes the number of sampling points used for the particle diameter and $\Delta D=\left(D_{\max}-D_{\min}\right)/\left(K-1\right)$.

Taylor series expansion of the Langevin function in Equation (1) and consolidating similar items on a frequency basis allow *M* to be expressed as:

(5) $$\begin{array}{cc} M\left(H_{ac}\right) & =\frac{1}{V_{T}}\sum_{k=1}^{K}n\left(D_{k}\right)V\left(D_{k}\right)\sum_{j=1}^{\infty }A_{2j-1}\left(H,D_{k}\right)\sin\left[\left(2j-1\right)\omega t\right]\Delta D\\ & =\sum_{j=1}^{\infty }\frac{1}{V_{T}}\sum_{k=1}^{K}n\left(D_{k}\right)V\left(D_{k}\right)A_{2j-1}\left(H,D_{k}\right)\Delta D\sin[\left(2j-1\right)\omega t],\\ & =\sum_{j=1}^{\infty }C_{2j-1}\left(H\right)\sin\left[\left(2j-1\right)\omega t\right] \end{array}$$

where the relationship between *C*_2*j*-1_ and *A*_2*j*-1_ can be expressed as:

(6) $$C_{2j-1}\left(H\right)=\frac{1}{V_{T}}\sum_{k=1}^{K}n\left(D_{k}\right)V\left(D_{k}\right)A_{2j-1}\left(H,D_{k}\right)\Delta D.$$

where *A*_2*j*−1_ and *C*_2*j*−1_ represent the (2*j*−1)-th harmonic amplitude of magnetization from single MNP with *D_k_* and ensemble of MNPs, respectively. As shown in Equation (6), the contribution of the MNP with *D_k_* to the amplitude of the harmonics is given by the factor *n*(*D_k_*)*V*(*D_k_*).

To estimate the core size distribution, *C*_2*j*-1_ was measured for *N* different amplitudes of the AC excitation field, i.e., *H_i_* (*i* = 1, 2, …, *N*). By introducing the *N* × 1 vector **C**_2*j*-1_ with component *C*_2*j*-1_(*H_i_*), the *K* × 1 vector **X** with component *n*(*D_k_*)*V*(*D_k_*), and the *N* × *K* matrix **B**_2*j*-1_ with component $\frac{1}{V_{T}}A_{2j-1}\left(H_{i},D_{k}\right)\Delta D$, the following equation can be obtained:

(7) $$\begin{array}{c} C_{2j-1}=B_{2j-1}X,\\ C_{2j-1}=\left[\begin{array}{c} C_{2j-1}\left(H_{1}\right)\\ C_{2j-1}\left(H_{2}\right)\\ \vdots \\ C_{2j-1}\left(H_{N}\right) \end{array}\right],\;X=\left[\begin{array}{c} n\left(D_{1}\right)V\left(D_{1}\right)\\ n\left(D_{2}\right)V\left(D_{2}\right)\\ \vdots \\ n\left(D_{K}\right)V\left(D_{K}\right) \end{array}\right],\\ B=\left[\begin{array}{cccc} A_{2j-1}\left(H_{1},D_{1}\right) & A_{2j-1}\left(H_{1},D_{2}\right) & \cdots & A_{2j-1}\left(H_{1},D_{K}\right)\\ A_{2j-1}\left(H_{2},D_{1}\right) & A_{2j-1}\left(H_{2},D_{2}\right) & \cdots & A_{2j-1}\left(H_{2},D_{K}\right)\\ \vdots & \vdots & \ddots & \vdots \\ A_{2j-1}\left(H_{N},D_{1}\right) & A_{2j-1}\left(H_{N},D_{2}\right) & \cdots & A_{2j-1}\left(H_{N},D_{K}\right) \end{array}\right]. \end{array}$$

For the case when the *J* odd harmonic amplitudes of magnetization are used for the estimation of the core size distribution, the following matrix equation can be obtained:

(8) $$\begin{array}{c} Y=BX,\\ Y=\left[\begin{array}{c} C_{1}\\ C_{3}\\ \vdots \\ C_{2J-1} \end{array}\right],\;B=\left[\begin{array}{c} B_{1}\\ B_{3}\\ \vdots \\ B_{2J-1} \end{array}\right]. \end{array}$$

The inversion problem can be solved numerically by using a mathematical technique such as nonlinear-non-negative least squares (NNLS) or singular value decomposition (SVD). The ill-posed matrix equation can be improved by the regularization method [13,16]. Because the magnetization response of small particles is linear, they may have no higher harmonics. Meanwhile, the amplitude of the harmonics decreases with increasing harmonics number. Therefore, we use only the 1st, 3rd, and 5th magnetization harmonics of the MNPs under different AC excitation field strengths for constructing the matrix equations, i.e., *J* is set to 3 in Equation (8). The 1st, 3rd, and 5th harmonic amplitudes of magnetization with *D_k_* can be given as:

(9) $$A_{1}\left(H_{i},D_{k}\right)=M_{s}\left(\frac{x}{3}-\frac{x^{3}}{60}+\frac{x^{5}}{756}-\frac{x^{7}}{8640}+\frac{x^{9}}{95040}+\cdots \right),$$

(10) $$A_{3}\left(H_{i},D_{k}\right)=M_{s}\left(\frac{x^{3}}{180}-\frac{x^{5}}{1512}+\frac{x^{7}}{14400}+\cdots \right),$$

(11) $$A_{5}\left(H_{i},D_{k}\right)=M_{s}\left(\frac{x^{5}}{7560}+\frac{x^{7}}{43200}+\cdots \right),$$

where

(12) $$x=\frac{\mu_{0}m_{k}H_{i}}{k_{B}T}.$$

By measuring *M* under an AC excitation field *H_i_*, the component *C*_2*j*-1_(*H_i_*) of vector **Y** can be obtained via a digital phase sensitive detection (DPSD) algorithm. The component *A_ik_* of vector **B** can be determined based on Equations (9)–(11), and thus the vector **X** with component *n*(*D_k_*)*V*(*D_k_*) can be obtained by solving the inverse problem given by Equation (8).

## 3. Simulation Results

Simulations were performed to verify the feasibility of estimating the core size distribution of MNPs via harmonic amplitudes of the magnetization under AC excitation. For the distributions, we assumed that *n*(*D*)*V*(*D*) obeyed a bimodal log-normal size distribution given by:

(13) $$n(D)V(D)=\sum_{k=1}^{2}\omega_{k}LN\left(D;\mu_{k},\sigma_{k}^{2}\right),$$

(14) $$LN\left(D;\mu_{k},\sigma_{k}^{2}\right)=\frac{1}{\sigma_{k}D\sqrt{2\pi }}\exp\left[-\frac{{\left(\ln D-\ln\mu_{k}\right)}^{2}}{2\sigma_{k}^{2}}\right],$$

where *μ* and *σ* are the mean and standard deviation, respectively. Table 1 and Table 2 list the parameters for different MNP samples with bimodal log-normal distributions.

**Figure 1:**
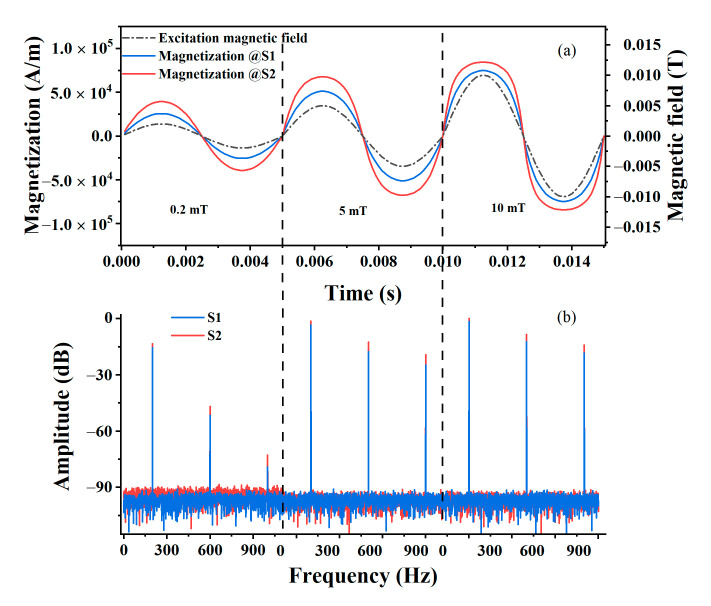
Magnetization of MNPs depends on the core size distribution. (**a**) Waveforms of MNP magnetization for different core size distributions. (**b**) Frequency spectrum. The sampling frequency was 1 MHz and the sampling cycle was 50. Waveforms of the magnetization at equilibrium for each excitation field amplitude are plotted in (**a**).

In the simulations, *D* ranged over 1–50 nm with a step size of 1 nm, and the AC excitation field had an amplitude over the range 1–10 mT, with a step size of 0.2 mT and a frequency of 200 Hz. The saturation magnetization of the MNPs was 210 kA/m. The magnetizations of MNP samples for different AC excitation fields and a fixed 297 K temperature were calculated via Equation (4). As shown in Figure 1, the core size distribution of the MNP sample had a significant effect on the magnetization.

In the simulations, *D* ranged over 1–50 nm with a step size of 1 nm, and the AC excitation field had an amplitude over the range 1–10 mT, with a step size of 0.2 mT and a frequency of 200 Hz. The saturation magnetization of the MNPs was 210 kA/m. The magnetizations of MNP samples for different AC excitation fields and a fixed 297 K temperature were calculated via Equation (4). As shown in Figure 1, the core size distribution of the MNP sample had a significant effect on the magnetization.

**Figure 2:**
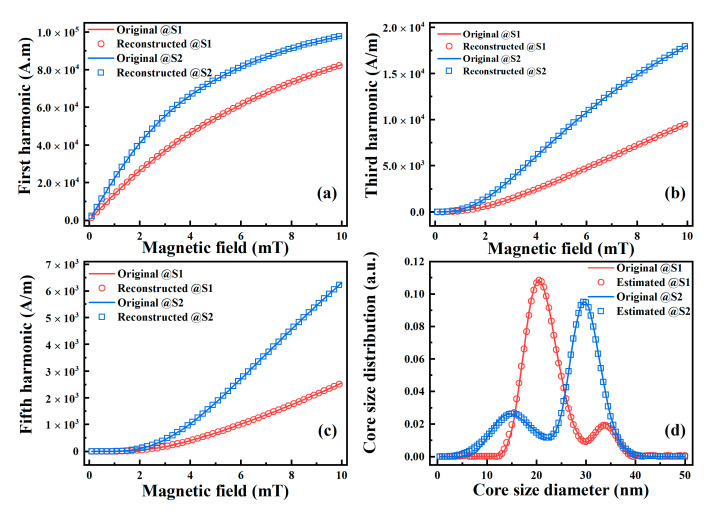
Simulation results of the (**a**) 1st, (**b**) 3rd, (**c**) 5th harmonics of MNP magnetization, and (**d**) the estimated MNP core size distributions using the harmonic amplitudes.

The amplitudes of the harmonics of the ensemble magnetization can be obtained via the DPSD algorithm for different AC excitation fields. The first, third, and fifth harmonic amplitudes of magnetization with *D_k_* were calculated via Equations (9)–(11), respectively, and the core size distribution was estimated by the SVD algorithm based on Equation (8). Figure 2d illustrates the estimated core size distributions for each MNP sample with different parameters. The solid lines indicate the original core size distributions, while symbols indicate the distributions estimated with harmonic amplitudes. There is excellent agreement between the estimated distributions and the original ones. Moreover, we used the estimated distributions to reconstruct the harmonic amplitudes of magnetization. As shown in Figure 2a–c, the first, third, and fifth harmonics of magnetization reconstructed by estimated distributions agree well with those of the original magnetization.

## 4. Experimental Results and Discussion

To verify the simulation results for the estimated core size distributions of MNPs via harmonic amplitudes of magnetization under an AC excitation field, three experiments were performed. Commercial MNPs (SHP-20, Ocean NanoTech, San Diego, CA USA) were used as samples. SHP-20 MNPs are iron oxide nanoparticles with carboxylic acid groups and an iron concentration of 5 mg/mL. The solvent of the sample is deionized H_2_O with 0.03% NaN_3_. The AC susceptibility was first evaluated within the frequency range 10–100 kHz to choose an available frequency without the effect of Brownian relaxation. Then we measured the magnetization harmonics of the MNP sample at different AC excitation fields and estimated the core size distribution via Equation (8). Finally, the distribution was estimated via the *M*–*H* curve method to compare the results with that based on magnetization harmonics.

### 4.1. Excitation Frequency without the Effect of Brownian Relaxation

**Figure 3:**
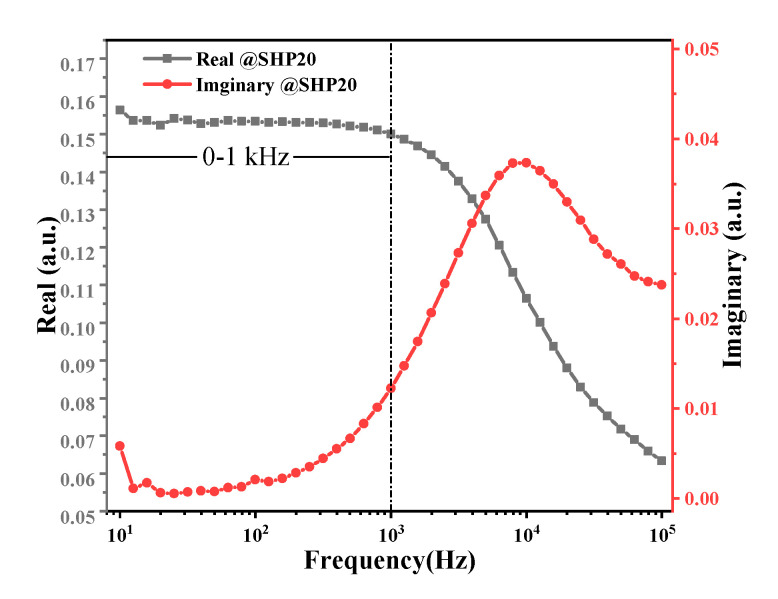
The AC susceptibility of SHP-20 vs. frequency.

To satisfy the condition of magnetization linearity, a weak excited magnetic field was applied at 0.1 mT/*μ*_0_ with a frequency ranging over 10–100 kHz. The AC susceptibility of the MNP sample is plotted in Figure 3. The imaginary part had a peak at frequency *f* = 1/(2*πτ_B_*), where *τ_B_* is the Brownian relaxation time. When the excitation frequency was less than 1 kHz, the real part of the AC susceptibility approached a constant value (DC susceptibility), which indicated that the effect of Brownian relaxation on the MNP magnetization response could be ignored at low frequencies. Note that the magnetization of noninteracting and identical MNPs under AC excitation fields with low frequencies can be described by the Langevin function.

### 4.2. Estimation of Core Size Distribution

The static *M*–*H* curve of the SHP-20 sample was acquired to determine the saturation magnetization and core size distribution. The saturation magnetization was determined at a static magnetic field strength of 1 T. The saturation magnetization of SHP-20 was 205 kA/m.

**Figure 4:**
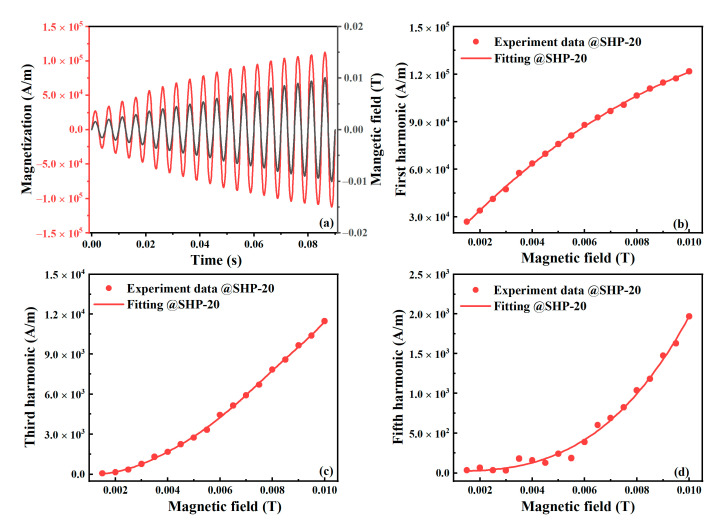
The (**a**) magnetization of MNP samples under different magnetic field excitations, and the (**b**) 1st, (**c**) 3rd, (**d**) 5th harmonics of MNP magnetization. The AC magnetic excitation field was 1.5–10 mT, with a step size of 0.5 mT at a frequency of 200 Hz. The experimental data at different magnetic field excitations was shown in one period.

A glass tube with the MNP sample was kept at a constant 297 K and placed at the geometric center of a solenoid coil for AC excitation. The excitation frequency was 200 Hz to avoid the effect of Brownian relaxation. The excitation amplitude was varied over 1.5–10 mT, with a step size of 0.5 mT. In this case, the effect of the Brownian relaxation time could be ignored, as shown in Figure 3. The magnetizations of SHP-20 under different excitation fields are plotted in Figure 4. Note that one cycle of the magnetization in an equilibrium state for each excitation field amplitude is plotted in Figure 4a for visual clarity. We can then obtain the 1st, 3rd, and 5th harmonic amplitudes via DPSD. In Figure 4b–d, the points show the experimental data of the harmonic amplitudes at different AC excitation field amplitudes, while the solid lines are polynomial curve fittings with a constraint of *M_i_* (*H* = 0) = 0.

The relationship between harmonic amplitudes and the excitation field is theoretically given by Equations (6)–(8), and thus the MNP core size distribution can be estimated. The range of *D* was set to 0.5–50 nm, with a step size of Δ*D* = 0.5 nm; i.e., *K* = 100. Using the harmonics data (AC magnetic field varied over 1.5–10 mT, with a step size of 0.5 mT), the matrix equation can be solved. However, the calculated results were not very good because of less harmonics data and a larger measurement error (noise) of the higher harmonics at low excitation fields. Therefore, we obtained more harmonics data under different excitation fields (1.5–10 mT) by fitting the curve. In this case, we used harmonic amplitudes data for excitation fields ranging over 1.55–10 mT, with a step size of 0.05 mT, i.e., *N* = 170. In this manner, each component of the coefficient matrix could be calculated from Equations (9)–(11). The estimated core size distribution of SHP-20 was obtained when we took seven of the largest singular values in the SVD method.

**Figure 5:**
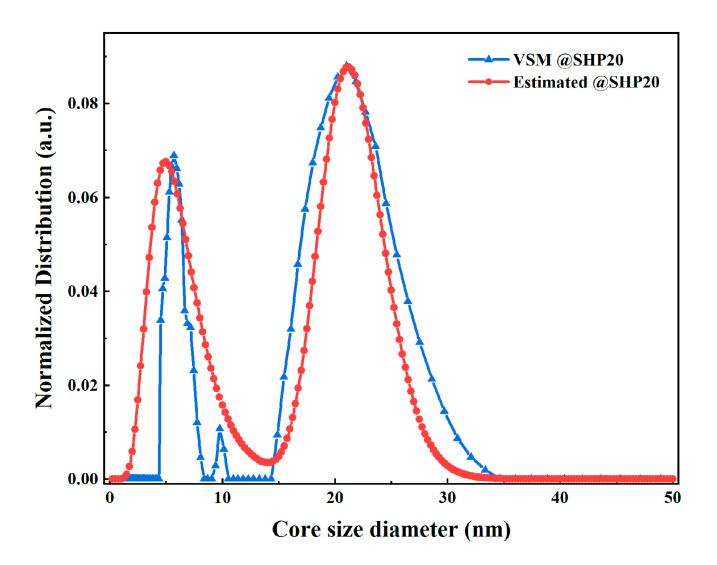
The core size distribution of SHP-20 estimated by harmonics amplitudes via the singular value decomposition (SVD) method (red) and *M–H* curves via the NNLS method (blue).

Figure 5 compares the core size distributions of SHP-20 estimated via magnetization harmonics and *M*–*H* curves. The results estimated via magnetization harmonics reasonably match those via *M*–*H* curves, and both have a peak value at 21 nm.

### 4.3. Comparison via Reconstruction of Magnetization and AC Harmonics

**Figure 6:**
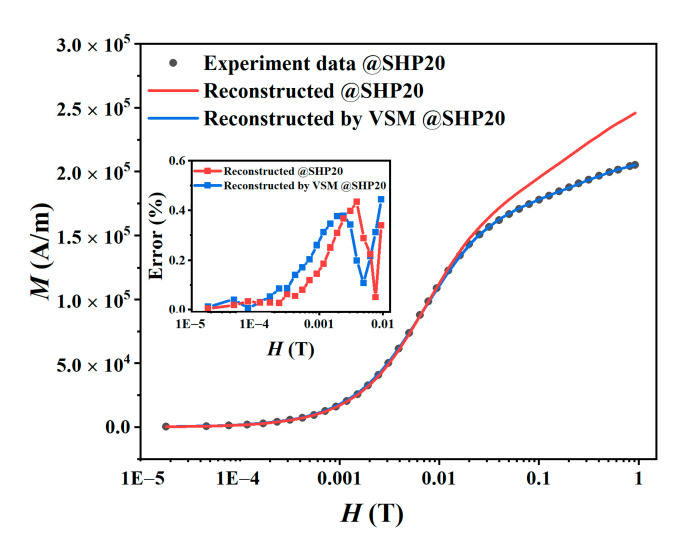
Experimental and reconstructed static *M–H* curves of SHP20. The illustration is the error between the experimental data and the reconstructed results.

**Figure 7:**
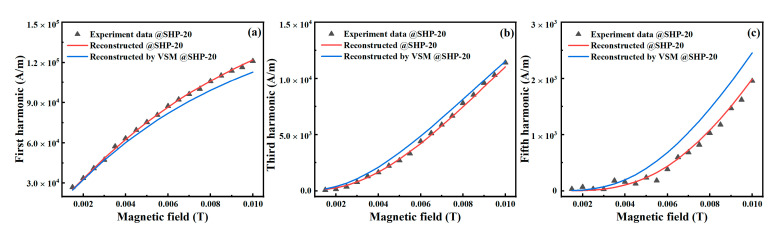
Experimental and reconstructed results of harmonic amplitudes. The points are experimental data, the red lines indicate analytical results reconstructed with the core size distribution estimated via harmonics amplitudes, and the blue lines represent that estimated by *M–H* curves. (**a**–**c**) are the 1st, 3rd, and 5th harmonics, respectively.

If the core size distribution of MNPs can be obtained, the magnetization *M* of the MNPs can be calculated from Equation (1), and we reconstructed the static *M*–*H* curves and the harmonic amplitudes of magnetization. The red and blue lines in Figure 6 and Figure 7 are the reconstructed results calculated with the core size distribution estimated by the harmonic amplitudes and the *M*–*H* curves, respectively. For the core size distribution estimated by the *M*–*H* curve, the reconstructed result of the *M*–*H* curve agrees well with the experimental data, although clear differences can be observed in the harmonic amplitudes. In contrast, for the core size distribution estimated by harmonic amplitudes, the reconstructed results of the *M*–*H* curve at low DC excitation fields and harmonic amplitudes agree well with the experimental data. These results indicate that the distribution of large core sizes can be more accurately estimated by harmonic amplitudes than by the static *M*–*H* curve.

However, as depicted by the red curve in Figure 6, a clear difference can be observed in the *M*–*H* curve between the experimental data and reconstructed result at large DC excitation fields. Hence, the estimation for smaller particles needs a higher AC excitation field to induce magnetization, especially for higher harmonics. Therefore, we discuss the effect of the strength of excitation fields on the estimated core size distributions.

### 4.4. Discussion

**Figure 8:**
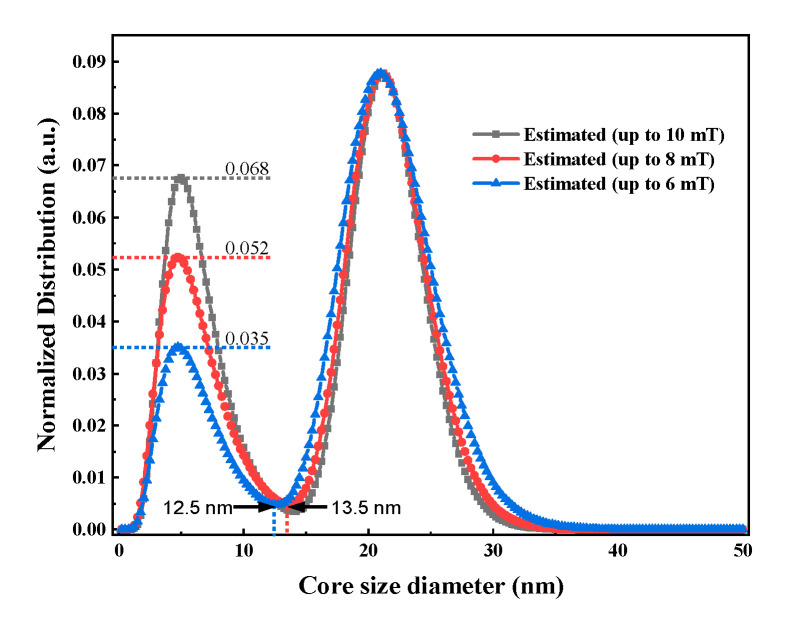
Core size distribution of SHP-20 estimated by magnetization harmonics with different excited magnetic field strengths.

In the experiments, the AC excitation field was 1.5–10 mT, with a step size of 0.5 mT and a frequency of 200 Hz. To evaluate the effect of excitation field strength, we used different ranges up to 6, 8, and 10 mT for the estimation of core size distributions. Figure 8 plots the estimated results under different ranges of excitation fields. All the estimated distributions have the same peak value of 21 nm. There is a small peak at 4.8 nm, but the densities at 4.8 nm are different. The density of the small peak increases with excitation magnetic field strength.

To quantitatively clarify the relationship between the maximum AC excitation field amplitude and the lower limitation of the estimated core size distribution, we discuss the parameter *x* given by Equation (12). When *x* < 1, the magnetization given by Equations (1)–(3) is approximated by *M*_s_*ξ*/3 = *M*_s_*x*sin(*ωt*)/3; i.e., its magnetization is in the linear regime and only the first harmonic magnetization can be observed for the most part, as shown in Equations (9)–(11). In this case, information on the core size distribution, which is included in *C*_2j-1_ in Equation (6), is lost. Consequently, the MNP distribution that satisfies *x* < 1 cannot be accurately estimated. The core sizes of MNPs with *x* = 1 are 18.5, 16.8, and 15.6 nm for maximum AC excitation field amplitudes *µ*_0_*H*_max_ = 6, 8, and 10 mT, respectively. As shown in Figure 8, there is a trough at 12–15 nm. Therefore, distributions of MNPs with core sizes larger than 15 nm can be accurately obtained for *µ*_0_*H*_max_ = 10 mT. As shown in Figure 8, estimated distributions of core sizes larger than 15 nm gradually deviate from that obtained for *µ*_0_*H*_max_ = 10 mT with decreasing *µ*_0_*H*_max_. Moreover, the densities at 4.8 nm are strongly affected and deviate from that obtained for *µ*_0_*H*_max_ = 10 mT.

A higher excitation field easily induces magnetization of small MNPs. However, it requires a higher current and a more complex system design, such as a resonant circuit to reduce the impedance. Moreover, the greater current will generate more heat, and changes in the temperature of the excitation coil will cause changes in its impedance characteristics, which will affect the stability of the AC excitation field.

In summary, the method of using harmonic amplitudes for the estimation of core size distributions has the advantage that large core size distributions can be more accurately and quickly estimated than by static *M*–*H* curves. Magnetization signals from large core MNPs are commonly used for biomedical applications. However, there is a limitation for small MNPs. The higher excitation field easily induces magnetization of small MNPs, which is needed for higher estimation accuracy of small core size distributions. Therefore, the excitation frequency and magnetic field strength are chosen carefully, depending on the time resolution and estimation error of the AC harmonic method.

## 5. Conclusions

An improved method for estimating core size distributions of MNPs was presented. It used an excitation frequency based on experimental AC susceptibility to avoid the effect of Brownian relaxation. The first, third, and fifth magnetization harmonics under different excitation field strengths were used to estimate the distributions, rather than using all the available magnetization harmonics at a single excitation magnetic field. The distributions can be obtained without assuming any distribution function. The harmonic amplitudes of the MNP magnetization reconstructed from the estimated distribution agree well with experimental results. Using this method, the distributions of large core sizes can be more accurately and quickly estimated than by the static *M*–*H* curve method. This is a competitive advantage in magnetic nanoparticle immunoassays. The improved method for estimating core size distributions is expected to optimize MNPs for various applications.

## Figures and Tables

**Table 1:** Parameters of core size distribution for magnetic nanoparticle (MNP) sample S1.

	*k* = 1	*k* = 2
Weight *ω_k_*	0.9	0.1
Geometric mean *μ_k_* (nm)	21	34
Geometric standard deviation *σ_k_*	0.16	0.06

**Table 2:** Parameters of core size distribution for MNP sample S2.

	*k* = 1	*k* = 2
Weight *ω_k_*	0.3	0.7
Geometric mean *μ_k_* (nm)	16	30
Geometric standard deviation *σ_k_*	0.3	0.1
